# 单孔和单操作孔胸腔镜技术在肺癌外科治疗中的临床效果分析

**DOI:** 10.3779/j.issn.1009-3419.2020.101.23

**Published:** 2020-07-20

**Authors:** 伟峰 徐, 春 徐, 成 丁, 俊 陈, 文毅 王, 军 赵, 畅 李

**Affiliations:** 1 215006 苏州，苏州大学附属第一医院胸外科 Department of Thoracic Surgery, The First Affiliated Hospital of Soochow University, Suzhou 215006, China; 2 214105 无锡，无锡市锡山人民医院胸外科 Department of Thoracic Surgery, Wuxi Xishan People's Hospital, Wuxi 214105, China

**Keywords:** 单孔, 胸腔镜, 单操作孔, 肺肿瘤, Uniport, Video-assisted thoracoscopic, Biport, Lung neoplasms

## Abstract

**背景与目的:**

近年来单孔胸腔镜肺癌根治术逐渐兴起，作为一种新的手术方式，其可行性及安全性尚未得到普遍认可。本研究拟探讨单孔胸腔镜肺癌根治术在治疗Ⅰ期至部分Ⅲa期肺癌病例中的可行性及安全性。

**方法:**

回顾性分析苏州大学附属第一医院胸外科同一治疗组在2018年5月-2019年3月连续进行的胸腔镜肺癌根治术患者的临床资料。排除部分不符合要求的病例后，根据手术方式分为单孔组（55例）和单操作孔组（87例），共142例。分析两组患者的临床数据，进行统计学分析。

**结果:**

142例患者均行肺叶切除+淋巴结清扫术，术后顺利出院，未有围手术期死亡。单孔组和单操作孔组比较，年龄、肿瘤位置、术后病理类型、肿瘤大小、肿瘤的病理性原发灶-淋巴结-转移（pathological tumor-node-metastasis, pTNM）分期的差异均无统计学意义（*P* > 0.05）。单孔组手术时间更短[(167.65±43.85) min *vs* (181.71±51.28) min]，术中出血量更少[(57.45±50.19) mL *vs* (87.47±132.54) mL]，术后引流管留置时间[(4.82±2.82) d *vs* (5.84±3.43) d]及术后住院天数[(6.91±3.88) d *vs* (7.74±3.87) d]更短，但差异无统计学意义（*P* > 0.05）。单孔组的术后总引流量显著低于单操作孔组[(1, 064.82±776.38) mL *vs* (1, 658.71±1, 722.38) mL]，术后24 h及72 h视觉疼痛评分明显更低[（4.73±0.73）分*vs*（5.25±0.74）分；（2.16±0.71）分*vs*（2.55±0.86）分]，差异有统计学意义（*P* < 0.05）。

**结论:**

在Ⅰ期至部分Ⅲa期肺癌病例中，单孔胸腔镜肺癌根治术安全可行。

## Abstract (en)

**Background and objective:**

In recent years, the technique of uniportal video-assisted thoracoscopic surgery has been developed. As a new surgical method, its feasibility and safety have not been generally recognized. The aim of this study is to review the technology in the treatment of lung cancer patients in stage Ⅰ to part of stage IIIa.

**Methods:**

The clinical data of patients accepted thoracoscopic resection of lung cancer by a same medical group from May 2018 to March 2019 in The First Affiliated Hospital of Soochow University were retrospectively analyzed. After remove of cases that did not meet the requirements, the patients were divided into uniportal group (55 cases) and biportal group (87 cases). The clinical data of the two groups were collected and statistically analyzed.

**Results:**

All the 142 patients underwent lobectomy and systemic lymph node dissection. There is no perioperative death in both groups. There was no significant difference in age, location of tumors, pathological type, size of tumors and pathological tumor-node-metastasis (pTNM) staging between uniportal group and biportal group (*P* > 0.05). The operation time [(167.65±43.85) min *vs* (181.71±51.28) min], the intraoperative bleeding volume [(57.45±50.19) mL *vs* (87.47±132.54) mL], the indwelling time of drainage tube [(4.82±2.82) d *vs* (5.84±3.43) d] and the hospital stay [(6.91±3.88) d *vs* (7.74±3.87) d] were less in uiportal group compared to biportal group, though no significant difference occurred (*P* > 0.05). The total drainage volume of uniportal group was significantly lower than that of biportal group [(1, 064.82±776.38) mL *vs* (1, 658.71±1, 722.38) mL], and the visual analogue score of 24 hours and 72 hours after operation [(4.73±0.73) points *vs* (5.25±0.74) points; (2.16±0.71) points *vs* (2.55±0.86) points] were lower in uniportal group (*P* < 0.05).

**Conclusion:**

Uniportal video-assisted thoracoscopic radical resection of lung cancer is safe and feasible for stage Ⅰ to part of stage IIIa lung cancer patients.

2018年全球范围内，所有恶性肿瘤疾病中，肺癌的发病率及死亡率均居第一位^[1]^。外科手术仍是治疗肺癌尤其是非小细胞肺癌的重要方法。传统肺癌手术需采用标准开胸后外侧切口，手术创伤较大。1992年，Lewis等^[2]^在全球率先报道了胸腔镜下肺叶切除术。手术伤口明显缩小，术后疼痛显著减轻，术后恢复快，并发症少，在全世界迅速开展起来。近10年，由Gonzalez^[3]^引领的单孔胸腔镜肺部手术在全世界广泛传播，能完成较为复杂的肺癌根治术术式。单孔胸腔镜手术切口更少，但胸外科医师对于该技术的安全性、可行性及肿瘤学长期效果方面，还存在不同程度的担忧，从而需要更多的研究。本研究回顾性分析了2018年5月-2019年3月苏州大学附属第一医院胸外科同一治疗组通过单孔胸腔镜及单操作孔胸腔镜两种技术完成肺癌根治术的病例，探讨单孔胸腔镜手术的安全性和可行性。

## 1 资料和方法

### 1.1 研究对象

本研究回顾性分析了苏州大学附属第一医院胸外科同一治疗组在2018年5月-2019年3月连续进行的胸腔镜肺癌根治术患者的临床资料。根据手术方式分为单孔胸腔镜肺癌根治术组（单孔组）和单操作孔胸腔镜肺癌根治术组（单操作孔组）。入选的病例需要符合以下标准：①术后常规病理证实为肺癌，且均为原发性肺癌；②术前检查提示患者无远处脏器转移，排除Ⅳ期肺癌患者；③术前检查提示患者主要脏器无显著功能障碍，肺功能可以耐受开胸手术；④术前患者均未接受新辅助化疗、放疗、靶向药物治疗等；⑤术前患者无慢性疼痛疾病、无感觉障碍等影响术后疼痛评分因素；⑥手术方式均为肺叶切除术加系统性肺门、纵隔淋巴结清扫术；⑦手术初始方案为单孔胸腔镜方式或单操作孔胸腔镜方式。此外，需要排除下列情况的病例：①采用胸腔镜下肺楔形切除方式的肺癌患者；②肺段切除术患者；③全肺切除术患者；④进入胸腔后发现胸腔广泛致密黏连、花费较长时间分离黏连者；⑤术前有较多基础疾病、胸廓严重畸形的患者；⑥同期行双侧肺部手术的患者。病例总数142例，其中单孔组55例，单操作孔组87例。患者基本临床信息见表 1，术后常规病理报告情况见表 2。

**1:** 临床基本信息 Basic clinical information

Items	Uniportal group (*n*=55)	Biportal group (*n*=87)	*t*/*χ*^2^	*P*
Gender			5.524	0.019
Male	18	46		
Female	37	41		
Age (Mean±SD, yr)	60.76±9.82	59.82±11.06	0.525	0.600
Tumor location			2.669	0.615
Right upper lobe	18	23		
Right middle lobe	9	9		
Right lower lobe	12	21		
Left upper lobe	11	21		
Left lower lobe	5	13		

**2:** 术后常规病理 Pathological reports after operation

Items	Uniportal group (*n*=55)	Biportal group (*n*=87)	*t*/*χ*^2^	*P*
Pathological type			3.112	0.683
Adenocarcinoma	49	76		
Squamous cell carcinoma	4	8		
Compound small cell carcinoma + adenocarcinoma	1	0		
Lymphoepithelioma-like carcinoma	1	1		
Adenosine carcinoma	0	1		
Sarcomatoid carcinoma	0	1		
Tumor size (Mean±SD, cm)	2.54±2.74	2.56±1.82	-0.039	0.969
pTNM stage			12.467	0.086
Ⅰa1	7	9		
Ⅰa2	25	25		
Ⅰa3	5	12		
Ⅰb	6	16		
Ⅱa	0	1		
Ⅱb	9	7		
Ⅲa	3	14		
Ⅲb	0	3		
The stage of pTNM followed the 8th Edition Lung Cancer Stage Classification. pTNM: pathological tumor-node-metastasis.				

### 1.2 麻醉及手术方法

全身麻醉，插双腔气管导管，术中采取健侧单肺通气。单孔组采用腋前线略偏后切口，长度一般为3 cm。如为上肺或中肺手术，选用第4肋间切口。如为下肺手术，选用第5肋间切口。应用一次性切口保护套保护切口，使用德国STORZ电视胸腔镜系统，10 mm 30°胸腔镜。如术前有病理证实为恶性，或影像学提示肿瘤靠近肺门，或肿瘤直径较大，无法行楔形切除，则直接行肺叶切除。否则先行肿瘤楔形切除，快速病理证实恶性再行肺叶切除。术中行肺门及纵隔淋巴结清扫。右侧需清扫第2R、4R、7、8、9组纵隔淋巴结，左侧需清扫第4L、5、6、7、8、9组纵隔淋巴结。手术结束前用罗哌卡因及利多卡因的混合液进行肋间神经阻滞。切口内留置多孔胸腔引流管一根，尖端放置至胸顶部，兼顾术后排气及排液。术后拔除胸腔引流管指征：咳嗽时无漏气，胸腔引流量24 h小于300 mL或引流液呈淡黄色，复查胸片提示余肺复张良好，听诊患侧呼吸音清晰。单操作孔组在利用单孔胸腔镜切口作为操作孔的基础上，腋后线第7或第8肋间再作一个1.5 cm左右的观察孔。胸腔镜一般放置于该孔，但根据手术操作情况可临时放置于操作孔内，观察孔放置内镜直线切割缝合器等手术器械以获得更好的手术操作角度。同样进行肋间神经阻滞。如切除下肺，术后在观察孔留置引流管一根；如切除上肺，操作孔另外留置一根引流管放置至胸顶部作为排气管。拔管指征同单孔胸腔镜手术。术后24 h及72 h测定患者视觉模拟疼痛评分：由患者自行指出疼痛位于一根无刻度线段的位置，检查者读出背面相对应的疼痛刻度数值。由于患者咳嗽及活动后疼痛会有明显加重，干扰疼痛评分，故要求患者指出稳定状态时疼痛指数。分为0分-10分。0分表示无疼痛，分数越高，表示疼痛程度越严重；1分-3分表示轻度疼痛；4分-6分表示中度疼痛；7分-10分表示重度疼痛。

### 1.3 统计学方法

采用SPSS 25.0统计学软件进行统计学分析，比较两组的性别、年龄、肿瘤大小、位置、病理类型、病理肿瘤原发灶-淋巴结-转移（pathological tumor-node-metastasis, pTNM）分期、术中出血量、淋巴结清扫个数、术后总引流量、引流管留置时间、术后住院时间、术后24 h及72 h视觉模拟疼痛评分、术后并发症情况等。计量资料结果用均数±标准差表示，采用*t*检验；计数资料采用*χ*^2^检验。*P* < 0.05为差异有统计学意义。

## 2 结果

### 2.1 手术情况

142例患者均顺利完成手术，无围手术期死亡，均行肺叶切除+淋巴结清扫术，其中两组各有2例进行了右中下肺叶切除术+淋巴结清扫术。所有病例术后常规病理均证实为肺癌。

### 2.2 两组性别差异比较

单孔组的女性患者更多，单操作孔组的男性患者更多。但两组之间的年龄、肿瘤位置等均差异无统计学意义（*P* > 0.05），具体数据详见表 1。两组的病理类型、肿瘤大小、pTNM分期均差异无统计学意义（*P* > 0.05）。具体数据见表 2。

### 2.3 两组淋巴结清扫数量比较

两组差异无统计学意义（*P* > 0.05）。单孔组的手术时间要短于单操作孔组，术中出血量也少于单操作孔组，术后引流管留置时间及术后住院天数短于单操作孔组，但差异无统计学意义（*P* > 0.05）。单孔组的术后总引流量，术后24 h及72 h模拟视觉疼痛评分均显著低于单操作孔组，差异有统计学意义（*P* < 0.05）。具体数据见表 3。

**3:** 两组手术及术后情况统计（Mean±SD） Statistics of data in operation and post-operation in two groups (Mean±SD)

Items	Uniportal group (*n*=55)	Biportal group (*n*=87)	*t*	*P*
Operative time (min)	167.65±43.85	181.71±51.28	-1.681	0.095
Intraoperative bleeding volume (mL)	57.45±50.19	87.47±132.54	-1.607	0.110
Number of lymph nodes dissected	15.73±7.87	16.17±7.62	-0.335	0.738
Postoperative drainage days	4.82±2.82	5.84±3.43	-1.845	0.067
Hospitalization days after operation	6.91±3.88	7.74±3.87	-1.238	0.218
Total drainage volume after operation (mL)	1, 064.82±776.38	1, 658.71±1, 722.38	-2.798	0.006
Simulated visual pain score of 24 h after operation	4.73±0.73	5.25±0.74	-4.159	0.000
Simulated visual pain score of 72 h after operation	2.16±0.71	2.55±0.86	-2.912	0.004

### 2.4 两组并发症数据比较

见表 4。单孔组有1例中转开胸，原因为肿瘤与血管及支气管关系紧密，胸腔镜下操作困难。其余并发症均通过保守治疗好转。单操作孔组中皮下气肿或气胸6例，其中2例再次置管。肺栓塞1例，行胸部增强计算机断层扫描（computed tomography, CT）检查诊断明确后请介入科消融治疗后好转。2例中转开胸，其中1例为术中意外损伤肺动脉导致大出血，1例为肿瘤与血管及支气管关系紧密，胸腔镜下操作困难。单操作孔组患者2例因术后渗血再次手术止血；并发乳糜胸2例，其中1例5 d后再次手术结扎胸导管治愈，另1例保守治疗26 d后好转，拔除胸腔引流管。单孔组无并发症患者比例要高于单操作孔组，但差异无统计学意义（*P* > 0.05）。

**4:** 两组术中及术后并发症情况 Intraoperative and postoperative complications in two groups

Items	Uniportal group (*n*=55)	Biportal group (*n*=87)	*χ*^2^	*P*
Atrial fibrillation	2	1		
Pulmonary infection	5	7		
Continuous air leakage for more than 5 d	2	4		
Conversion to thoracotomy	1	2		
Pulmonary embolism	1	1		
Obvious subcutaneous emphysema or pneumothorax	1	6		
Re-operation for hemostasis	0	2		
Chylothorax	0	2		
Total	12 (10 patients)	25 (23 patients)		
Non-complication patients	45 (81.8%)	64 (73.6%)	1.287	0.257

## 3 讨论

近年来肺癌患者在胸外科患者中的比重越来越高，手术治疗仍然是肺癌治疗最为重要的治疗方式。与传统开胸手术相比，胸腔镜下肺癌切除术治疗Ⅰ期-Ⅱ期肺癌具有更少的术中失血量、更少的术后并发症、更短的术后住院时间，而且提高了5年总生存期^[4]^，近年来成为肺癌手术的主要手术方式。单孔胸腔镜肺癌切除术则进一步减少了手术切口。有研究^[5]^表明，和三孔胸腔镜手术相比，单孔胸腔镜手术术中出血量、术后引流量更少，术后带管时间及住院时间更短，术后疼痛更轻微，但清扫淋巴结无差异。此外，接受单孔胸腔镜肺癌切除术的患者，术后总体生理心理健康程度均优于接受三孔胸腔镜手术的患者^[6]^。也有学者专门分析了多篇文献，发现相比多孔胸腔镜手术，单孔胸腔镜手术可能在减少术后72 h内疼痛方面有一定的临床效果，但并没有达到统计学差异^[7]^。

单孔胸腔镜手术只有一个切口，合适的切口更有利于术中暴露及操作，反之，则可能增加手术难度，甚至需要额外增加或延长切口。我们选择右侧腋前线略偏后切口，左侧则略微更偏后一些，以避开心脏。该切口的优势在于靠近肺门，有利于肺门重要结构的观察及解剖。而且此处肋间隙较为宽大，便于器械进出，胸部手术选择靠前的伤口比靠后的伤口更不易导致术后慢性疼痛^[8]^。手术者及扶镜手均站在患者的腹侧，为避免扶镜手对术者造成干扰，我们的经验是，术者和扶镜手站位前后稍错开，术者在前，扶镜手在后，不影响术者左右小范围移动。术者双上肢位于扶镜手上肢上方，保证术者操作平面和扶镜手扶镜平面错开，两者都能不受影响的进行操作。胸腔镜身放置于切口的最后方，对于侧卧的患者而言，相当于放置于“最高处”，操作器械均从其下方进入。这样比较符合人体“眼高手低”的生理特点，操作更为自然。使用线绳绕过胸腔镜身，固定于手术单，给扶镜手提供一个人为的支点，减轻扶镜手工作强度。有时候因为角度或空间原因，内镜下切割缝合器要通过组织间隙到达预定位置较困难，又不能像多孔胸腔镜手术那样转换至另外切口操作。我们的经验是在需要插入内镜下切割缝合器的组织间隙穿过一根硅胶引流管来引导，再将硅胶引流管和切割缝合器相连接，这样可以很轻松地把切割缝合器送到指定位置，既安全又方便。

两组患者在手术结束前均进行肋间神经阻滞，我们选用罗哌卡因及利多卡因的混合液。前者是一种新型长效局麻药物，半衰期长，不易引发心脏毒性。后者起效快，弥散力强。两者结合使用，快速起效，作用持久。研究认为术中进行肋间神经阻滞可有效减轻术后早期疼痛^[9]^，并且罗哌卡因肋间神经阻滞还可以改善术后早期认知功能障碍^[10]^。胸部手术后24 h内疼痛最为剧烈，肋间神经阻滞能在术后早期发挥作用，相比硬膜外镇痛，更有利于患者术后早期活动，有效咳嗽排痰，减少术后肺部感染、肺不张、深静脉血栓等并发症，缩短住院时间^[11]^。

本研究中，单孔组术后疼痛明显小于单操作孔组。原因分析如下：①单操作孔组比单孔组要多出一个腋后线的切口，手术影响了两处肋间，加重了疼痛；②腋后线切口处肌肉相对腋前线更厚，肋间隙更窄，手术操作时胸腔镜身对切口处肋间神经卡压更明显；③单操作孔组行上叶切除的病例留置两根胸腔引流管，也在一定程度上加重了疼痛。有荟萃分析^[12]^表明，肺叶切除术后单根胸腔引流优于双根胸腔引流，体现在可以减少术后疼痛、术后引流时间及住院时间。此外，单根引流管并不增加术后并发症的风险，也不增加术后需再次引流的风险。我们建议术后尽早拔除胸管。有研究^[13]^证实胸管拔除后可以减少疼痛，并改善肺功能，尽早拔除胸管可以促进患者快速康复。胸管插入对肋间神经有明显损伤，可加重术后疼痛^[14]^。多项研究^[15, 16]^均证实单孔胸腔镜手术比双孔、多孔胸腔镜手术后疼痛更轻微。肺叶切除术后患者的疼痛控制非常重要，术后明显的疼痛可导致患者不愿活动，不能配合进行有效咳嗽排痰，增加术后肺不张、肺部感染、深静脉血栓的风险^[17]^。随着人们生活水平的逐步提高，以及肺癌患者越来越低龄化，这部分患者对手术后切口的美观、术后社会功能及整体生活质量有更高的要求。单孔胸腔镜术后胸壁仅有一个长约3 cm的切口，显然比单操作孔胸腔镜术后两个切口更为美观。在一组接受单孔胸腔镜手术的351例年轻气胸患者中，85%的患者对术后疤痕的美观度表示满意^[18]^。有研究^[19]^证实，与接受三孔胸腔镜手术相比，接受单孔胸腔镜手术者术后3个月整体生活质量明显更高，对切口更满意，切口麻木发生率也更低。

两组的手术时间和术中出血量无明显差异，和一些研究结果^[15, 20]^相同。两组的肿瘤大小、清扫淋巴结数量、肿瘤位置、病理类型、术后肿瘤分期均无明显差异，可以认为两组的手术难度基本相当。单孔胸腔镜肺癌根治术在我科开展时间晚于单操作孔肺癌根治术，但手术时间无明显差异，考虑我科已经过单孔胸腔镜手术学习曲线。一般认为，单孔胸腔镜肺癌根治术的学习曲线在30例左右^[21-23]^。随着手术病例数的进一步增加，手术时间可能继续缩短。单孔胸腔镜手术的优势在于视野方向和操作方向基本一致，这一点和传统开放手术非常类似。在适应了单孔胸腔镜手术的单一切口后，术者能够更自然、更方便地进行手术操作，从而缩短手术时间。有研究^[24]^表明，相比多孔胸腔镜手术，外科医生在进行单孔胸腔镜手术时可以保持更为中性舒适的身体姿势，颈椎的弯曲及旋转也明显改善。Gonzalez等^[25]^建议胸腔镜身与操作器械之间至少要有2 cm的距离，如果短于这个距离，器械之间互相干扰的可能性会大大增加。我们的切口长度约3 cm，在切口尽可能小和减少器械干扰之间取得了平衡。有研究^[26]^认为，对于非小细胞肺癌患者（部分Ⅰ期，全部Ⅱ期、Ⅲa期）进行系统性淋巴结清扫比淋巴结采样能延长生存期。有荟萃分析^[27]^表明，系统性淋巴结清扫相比淋巴结采样并没有增加并发症。两组所有患者均接受了淋巴结清扫术，如果为了追求“微创”而牺牲手术质量无疑是得不偿失的。

单孔组术后引流天数及术后住院天数均短于单操作孔组，但差异无统计学意义，既往研究^[15, 20]^也得出同样结果。有荟萃分析^[28]^表明单孔胸腔镜术后住院时间及引流时间较多孔胸腔镜术后显著下降。我们的研究已经显示单孔组较单操作孔组有一定优势，如能积累更多的病例数，可能有更明确的证据。本研究还显示，单孔组的术后总引流量明显低于单操作孔组。分析原因，术后两个切口渗出多于单孔组的一个切口，且增加的腋后线切口肌层较厚，缝合时不易全层兜底缝合，增加术后渗血及渗液量；单操作孔组引流时间似乎更长，部分患者留置2根胸管，可能造成更多的引流液；单操作孔组2例乳糜胸也明显增加了引流液。可以认为，单孔组术后恢复可能更快一些，这有助于降低患者医疗费用，降低平均住院天数，节省医疗资源。

两组主要的术后并发症是气胸、皮下气肿、持续漏气和肺部感染。手术结束前胸腔注水，观察余肺漏气情况尤为重要，可以采用缝合、医用胶水来处理漏气^[29]^。对明显肺气肿的患者，可在切割缝合器上加用垫片来防止漏气。单孔组有1例，单操作孔组有2例中转开胸手术，中转率分别为1.81%和2.30%。单孔组手术并发症并未比单操作孔组增加，因此，单孔胸腔镜肺癌根治术是安全的。

综上所述，单孔组相比单操作孔组具有明显更低的术后引流总量，术后疼痛明显更轻微，并且两组清扫淋巴结的数量无明显差异。尽管两组的手术时间、术中出血量、术后引流时间、住院时间、术后并发症均无明显差别，但单孔组相比单操作孔组相对更有优势。相信随着病例数的进一步增加，手术技术的不断进步，单孔组能有更明显的优势。因此，我们认为在Ⅰ期至部分Ⅲa期病例中，单孔胸腔镜肺癌根治术安全可行，具有术后疼痛小、恢复可能更快、切口美观的优点，是一项值得推广和发展的技术。值得注意的是，本研究仅涉及围手术期短期指标，对术后患者长期生存率、长期生活质量等方面没有涉及。这些都有待于今后进一步的研究。
